# The *Anabaena *sensory rhodopsin transducer defines a novel superfamily of prokaryotic small-molecule binding domains

**DOI:** 10.1186/1745-6150-4-25

**Published:** 2009-08-14

**Authors:** Robson F De Souza, Lakshminarayan M Iyer, L Aravind

**Affiliations:** 1 National Center for Biotechnology Information, National Library of Medicine, National Institutes of Health, Bethesda, MD 20894, USA

## Abstract

The *Anabaena *sensory rhodopsin transducer (ASRT) is a small protein that has been claimed to function as a signaling molecule downstream of the cyanobacterial sensory rhodopsin. However, orthologs of ASRT have been detected in several bacteria that lack rhodopsin, raising questions about the generality of this function. Using sequence profile searches we show that ASRT defines a novel superfamily of β-sandwich fold domains. Through contextual inference based on domain architectures and predicted operons and structural analysis we present strong evidence that these domains bind small molecules, most probably sugars. We propose that the intracellular versions like ASRT probably participate as sensors that regulate a diverse range of sugar metabolism operons or even the light sensory behavior in *Anabaena *by binding sugars or related metabolites. We also show that one of the extracellular versions define a predicted sugar-binding structure in a novel cell-surface lipoprotein found across actinobacteria, including several pathogens such as *Tropheryma*, *Actinomyces *and *Thermobifida*. The analysis of this superfamily also provides new data to investigate the evolution of carbohydrate binding modes in β-sandwich domains with very different topologies.

Reviewers: This article was reviewed by M. Madan Babu and Mark A. Ragan.

## Introduction

In the past decade membrane-embedded photoactive retinylidene-containing rhodopsins have been identified in several bacteria [1]. One such rhodopsin from the cyanobacterium *Anabaena*, which utilizes a chlorophyll-based photosynthetic apparatus, was proposed to function as a sensory rhodopsin, as opposed to being a proton pump [2-4]. Inference of a sensory function for the *Anabaena *sensory rhodopsin (ASR) was based on several features [2], including its co-transcription with a 14 kDa protein with which it was shown to physically interact. This co-transcribed 14 kDa protein has been proposed to function as an analog of the G-protein signal transducers associated with the animal visual rhodopsins [2,5] and was accordingly named ASRT ("Anabaena sensory rhodopsin transducer"). The solution of the three-dimensional structure of ASRT showed that it formed a tetrameric structure which was believed to mimic the G-β subunit of the animal visual rhodopsin [5]. However, it was observed that orthologs of ASRT were found in other bacteria, such as *Thermotoga maritma *[5], whose genomes do not encode any rhodopsin homolog. This observation raised questions regarding the more general function of the ASRT proteins and also its actual role in connection to ASR in *Anabaena*. This prompted us to systematically investigate ASRT homologs with the view of gleaning new functional information regarding this family by means of comparative genomics analysis. As a consequence we show that ASRT defines a novel family of prokaryotic small-molecule-binding domains that function in a wide range of contexts, predominantly as a carbohydrate sensor.

## Results and discussion

### Detection of novel ASRT homologs

The 3D structure of ASRT (PDB: 2II7) shows that the entire protein is comprised of a single globular domain with an eight-stranded β-sandwich fold [5]. All previously reported homologs of ASRT are similarly sized single-domain proteins (completely congruent to the PFAM alignment DUF1362 [6]) from various prokaryotes. To identify potential previously undetected homologs we initiated a series of sequence profile searches with known representatives of ASRT from different bacteria. A PSI-BLAST search with the *Thermotoga maritma *ASRT ortholog (PDB: 1NC7) against NR database detected segments of putative secreted amidases from certain chloroflexi with e-values of borderline significance (e = 0.013) and then converged. These alignments corresponded to regions that did not overlap with the amidase catalytic domain and did not correspond to any previously described domains. These segments in the amidases were also independently detected in a search with the ASRT alignment as a HMM against the NR database using the recently released HMMER3 program (e = .024). Despite their borderline e-values, the alignment with the ASRT starting point extended throughout the entire globular domain in the latter and showed conservation of structurally critical hydrophobic residues. To test this relationship we performed a search with the above-detected sequence segments from the amidases against a library of HMMs derived from PDB structures as seeds using HHpred, which performs profile-profile comparisons, and recovered the *Anabaena *ASRT and its *Thermotoga *orthologs as the best hits with significant e-values (E = 10^-3^). Likewise, a reciprocal PSI-BLAST search initiated with one of these regions detected in the amidases (residues 373-471 of gi:148657162; *Roseiflexus *sp. RS-1) identified *Anabaena *ASRT and its *Thermotoga *orthologs (e-values = 7*10^-3^, iteration 2) and in subsequent iterations retrieved all known ASRT homologs. This search also detected multiple repeats of the segment related to ASRT in the *Roseiflexus *amidase-domain-containing protein. In further transitive searches using representatives of these additional segments as query (e.g. 148270795, 679-789), we retrieved, in addition to the previously identified homologs, sequences from actinobacteria such as *Tropheryma whipplei*. Reciprocal PSI-BLAST searches with representatives of the segments detected in these actinobacterial sequences (e.g. gi:28493092) retrieved several other actinobacterial sequences together with representatives of sequences found in the initial searches (see Additional file 1 for details). None of these newly found sequences was recovered by the PFAM model DUF1362 or has been previously reported as being related to ASRT. The secondary-structure prediction for the newly detected sequences with significant relationship to ASRT suggested an all-β fold congruent with that determined for ASRT and it *T. maritima *ortholog. Taken together, these investigations indicated that ASRT and its cognates from other prokaryotes belonged to a larger superfamily of homologous domains, which might occur in more than one copy and in combination with other domains in a polypeptide (see below). Hereinafter, we refer to this domain as the ASRAH (for ***A**nabaena ***s**ensory **a**hodopsin- **a**ssociated omology) domain.

### Classification and structural analysis of ASRAH domains

**Figure 1:**
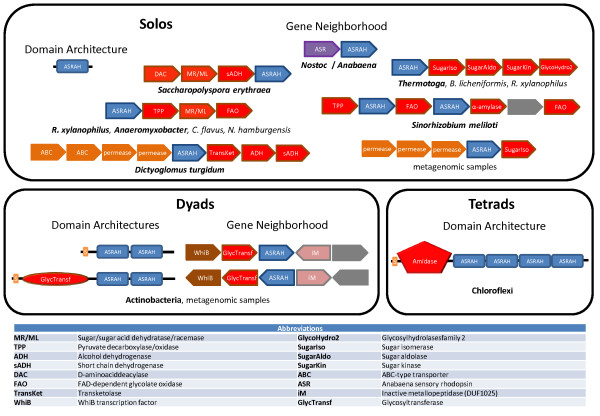
**Domain architectures and conserved gene neighborhoods of ASRAH homologs**. Gene neighborhoods are labeled by the organisms or taxonomic groups in which they are detected, of which the prototype species is depicted in bold letters. Complete descriptions for the gene abbreviations used in gene neighborhood and operon depictions are provided in the key below. The signal peptides in the actinomycete protein with two ASRAH domains are accompanied by a lipobox-like cysteine. Colors denote different functional classes, e.g. red represents enzymes or enzymatic domains, orange transporters, purple sensors. For a complete list of architectures and gene neighborhoods see Additional file 1.

**Figure 2:**
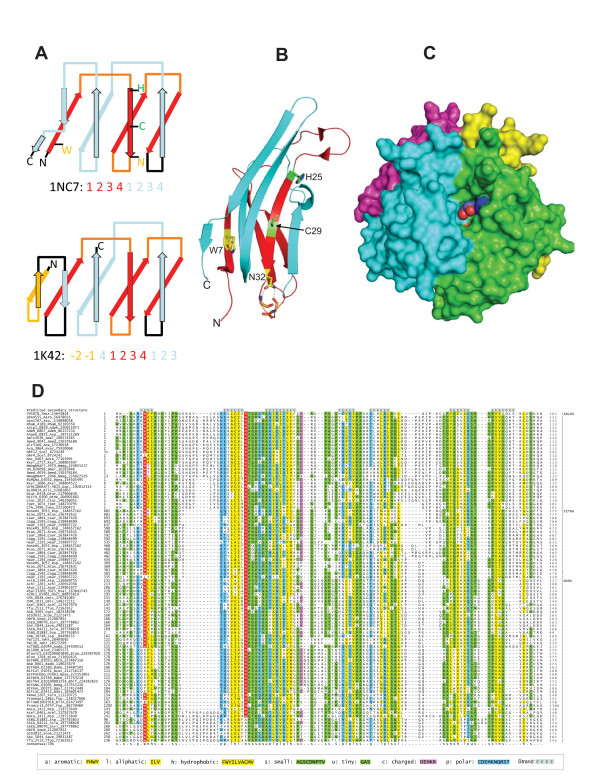
**Conserved structural and sequence features of the ASRAH domain**. A) Topology diagrams of *Thermotoga *TM1070 and the *Rhodothermus marinus *carbohydrate-binding module (CBM4-2) present in xylanase 10A. Equivalent strands are colored similarly. Numbers below each topology diagram illustrate the strand order. Note the circular permutation in the CBM4-2 domain. Additional N-terminal strands characteristic of CBM4-2 are colored orange. B) Cartoon representation of *Thermotoga *ASRAH homolog TM1070 (PDB: 1NC7), with key residue side-chains represented as sticks. Predicted ligand-binding residues are marked in green (H25 and C29), and other conserved residues (W7 and N32) in yellow. C) Surface model of the TM1070 tetramer, with the conserved histidine (H25) and cysteine (C29) of chain A marked in blue and yellow respectively. The 1,2-ethanediol molecule detected in the ligand-binding pocket of *Thermotoga *TM1070 is rendered as spheres. D) Comprehensive multiple sequence alignment of the ASRAH domain. Proteins are represented by their gene names, species abbreviations and gis. The coloring reflects the consensus at 70% conservation and highly conserved residues are shaded in red. Consensus abbreviations are listed in the lower panel. Refer to the Additional file 1 for species abbreviations.

Based on clustering with BLAST scores and domain architectural features the ASRAH domains can be divided into 3 families with distinctive phyletic patterns (see Additional file 1, Table S1 and Fig. 1 for details). The first of these, defined by the stand-alone, intracellular versions of the domain similar to ASRT, are found in addition to *Thermotoga *and *Anabaena *in some actinobacteria, proteobacteria, firmicutes, *Chthoniobacter flavus *(verrucomicrobia), the extremely thermophilic *Dictyoglomus *[7] as well as the archaeon *Natrialba magadii*. The second family is present in secreted proteins, always fused to an amidase domain, and is predominantly found in photosynthetic bacteria such as *Roseiflexus, Chloroflexus and Herpetosiphon*. The third family, also secreted, is found mainly in actinobacteria and is defined by unique N- and C-terminal conserved extensions flanking the ASRAH domains. A multiple sequence alignment of the ASRAH domain was generated by editing a preliminary alignment produced by the KALIGN program based on PSI-BLAST HSPs and secondary structure predictions (Fig. 2). Examination of the structures reveals that key structural elements of the *Anabaena *representative have been mutated to obtain its crystal structure. As consequence there are major distortions in this structure and it is unlikely to represent the native condition of the ASRAH domain. Hence, we continued further analysis using the *Thermotoga *ASRT structure determined as part of the Structural Genomics initiative (1NC7). Superposition of the secondary structure of this protein onto the alignment of the ASRAH superfamily shows that the sequence conservation is comprised predominantly of hydrophobic residues defining the core that stabilizes the β-sandwich. However, there are a few residues that distinguish this fold from other previously characterized β-sandwich folds. These include: 1) a well-conserved tryptophan, usually following a polar residue, present at the start of the first strand. This tryptophan appears to be central to a hydrophobic interaction required to hold the two β-sheets of the sandwich together (Fig. 2). 2) A nearly absolutely conserved asparagine located at the end of the second β-strand. The asparagine forms two hydrogen bonds with the backbone carbonyls of the residues 2 and 4 positions downstream from it. Further, there are no gaps in this part of the alignment suggesting that this conserved asparagine helps in stabilizing the characteristic tight turn between strand 2 and 3 of the structure.

An examination of the structure of the ASRAH domain suggests that the β-sandwich is composed of two internal repeats of four strands each - the first, third and fourth strand of each repeat are in one sheet of the β-sandwich while the second is in the opposite sheet. This topology distinguishes the ASRAH domain from other structurally comparable β-sandwich domains such as those found in the β-galactosidase [8] (shows evidence for independent duplication and lacks the C-terminal strand relative to the ASRAH β-sandwich) and the carbohydrate binding module CMB4 from the thermostable xylanase from *Rhodothermus marinus *[9] (which has additional N-terminal strands and displays a circular permutation of the last strand relative to the ASRAH β-sandwich; see Fig. 2). The structure shows that the solo ASRAH domain from *Thermotoga *exists as a tetramer with each unit containing a deep cleft that could potentially form a binding-pocket on one face of the sheet. In two of the monomers in the structure we observed that the pocket contains a 1,2-ethanediol molecule (Fig. 2), but contains considerable room for a much larger molecule such as a pentose or a larger sugar. Interestingly, the binding cleft is lined by two residues from the second strand containing the SHEshChhN signature (where "h" is a hydrophobic and "s" a small residue), which is nearly absolutely conserved in the ASRT-like family. The H and the C from this signature are seen to contact the 1,2-ethanediol in the structure, suggesting that they might be key to binding a ligand in this family. While this strand is well-conserved in the other families, these families possess their own distinctive residues instead of the H and C. This suggests that though they are likely to contain a similar cleft as the ASRT-like family, they might bind distinct ligands. Further, the walls of this cleft are formed in part by the dimer interface (Fig. 2). In line with this, we found that the ASRAH domains in the other families always occur either as dyads (the actinomycete family, Fig. 1) or as tetrads (the amidase associated versions, Fig. 1), suggesting that they might all have the potential to form homo- or hetero- tetrameric structures.

A ligand-binding pocket on one face of the sheet is particularly common in other β-sandwich domains, especially those involved in carbohydrate binding - e.g. the CMB4 of xylanases and the DOMON domain. However the ASRAH domain differs from the former in having its predicted-ligand pocket on the opposite face relative to the CMB4 domains and in being topologically very distinct from the ten-stranded DOMON domains [10].

### Domain architectures and gene neighborhoods point to a carbohydrate-binding role for the ASRAH domain

We sought to obtain further evidence for the ligand-binding role of the ASRAH domains based on their domain architectures and predicted operonic associations. The predicted gene neighborhoods of the solo versions of the ASRAH domain show multiple associations with genes encoding various carbohydrate metabolism proteins. One gene neighborhood conserved across many phylogenetically distant bacteria, including *Thermotoga*, combines a gene for the solo ASRT-like ASRAH proteins with genes encoding a sugar isomerase, a sugar aldolase and a sugar kinase. Another widely distributed bacterial conserved gene neighborhood combines the solo ASRT-like gene with a cluster of genes encoding an ABC transporter system specific for monosaccharide (Fig. 1). ASRT-like genes are also frequently combined in operons with genes encoding other potential sugar metabolism-related enzymes such as amylases (e.g. *Sinorhizobium*), pyruvate decarboxylase/oxidase, a sugar/sugar acid dehydratase/racemase and a FAD-dependent oxidoreductase related to the glycolate oxidase (Fig. 1). Interestingly, in the actinobacterium *Rubrobacter xylanophilus *two paralogous copies of ASRT orthologs are each associated with either the isomerase, aldolase and kinase gene neighborhood or the pyruvate decarboxylase/oxidase, dehydratase/racemase neighborhood. Thus, these linkages strongly support the ASRT-like domains regulating distinct sugar metabolism process in *cis*. The amidase domain found fused to the ASRAH domain is of the N-acetylmuramoyl-L-alanine amidase, which is consistent with a potential role for the ASRAH domain in binding elements of peptidoglycan. The cell-surface versions found mainly in the actinomycetes are predicted to be lipoproteins because their N-terminal region contains a conserved signal peptide sequence followed by an absolutely conserved cysteine reminiscent of the "lipobox" [11]. They additionally contain an internal cysteine, just N-terminal to the ASRAH domain (Fig. 2), which could form the site of a second distinct lipid modification. Their predicted gene-neighborhood context, which is conserved in practically all actinomycetes encoding such an ASRAH domain protein, shows a tight linkage to a transcription factor of the WhiB family, a glycosyltransferase and an inactive version of a zincin-like metallopeptidase. In some organisms the glycosyltransferase and the ASRAH domain containing protein are fused into a single polypeptide (*Frankia *sp. CcI3) supporting the functional linkage between these proteins. Thus, these cell-surface proteins also display contextual connections that are compatible with them binding sugars. It is likely that they are lipid-modified themselves and form an actinobacteria-specific cell-surface structure by binding a polysaccharide created by the linked glycosyltransferase or recruiting the same enzyme to a precursor polysaccharide.

Thus, the contextual evidence, combined with the structural evidence for ligand-binding, favors the ASRAH domain being a sugar-binding domain, in the manner of many other β-sandwich structures. This is not unexpected for the secreted or cell-surface versions of the ASRAH domain because it is consistent with the function of many other extracellular β-sandwich structures in binding cell-surface polysaccharides, which are parts of the peptidoglycan, glycoprotein and other polymeric carbohydrate layers that decorate prokaryotic cell surfaces. However, the intracellular standalone ASRAH domains are somewhat functionally unexpected because they appear to represent a novel regulatory mechanism - a potential sugar-sensor that might influence the function of other enzymes or sugar transporters encoded by the conserved gene-neighborhood via physical interactions with them depending on sugar concentrations. In light of this, we propose that the unusual linkage to the rhodopsin, which is observed only in the cyanobacterium *Nostoc*, might represent a novel mechanism in which the behavior of a light sensor (ASR) is influenced via interaction with an intracellular sensor of a sugar or a related metabolite (ASRT). It is plausible that this interaction might have a role in phototaxis in response to intracellular nutrient status.

### General discussion

Our investigation demonstrates that ASRT belongs to a superfamily of domains predicted to bind small molecules, most likely sugars, in both extracellular and intracellular locations in various bacterial and few archaeal proteins. On one hand the presence of sugar binding capability in a β-sandwich scaffold is hardly unprecedented for domains found in extracellular or cell-surface proteins. Nevertheless, the identification of such a potential sugar-binding element in a novel cell-surface lipoprotein is of interest especially given its presence across actinobacteria, including several pathogens such as *Tropheryma*, *Actinomyces *and *Thermobifida*. On the other hand, the identification of such a function in an intracellular context is of interest especially because the tetramer-forming standalone versions are predicted to regulate a diverse range of sugar metabolism operons or even the light sensory behavior in a cyanobacterium. This observation points to a previously unreported mechanism of regulation by a potential standalone small-molecular sensor that probably occurs at the level of protein-protein interactions rather than via the sensor domain of a one-component system transcription factor. In this respect such a regulatory process is closer to the allosteric regulation by small molecules.

While "sideways" ligand binding via one of the exposed sheets has been observed in several β-sandwich scaffolds (e.g. DOMON domain and other carbohydrate-binding domains [12]), the exact mode of binding is not established for all such folds. The analysis of the ASRAH domain presented here identifies the binding site for one more of these β-sandwich scaffolds. The proposed binding site for this superfamily of domains reinforces an observation that presents an interesting evolutionary conundrum: though several β-sandwich scaffolds bind ligands in a "sideways" fashion, their topologies greatly differ from each other. This leads to the question as to whether there was repeated convergent evolution of the "sideways" binding mode in various β-sandwiches or whether it represents an ancestral binding mode for the β-sandwich scaffolds, which was preserved despite the extensive topological rearrangements occurring as consequence of duplication of internal units or accretion of additional strands. Hence we hope that these observations would also contribute to the more general understanding of evolution of ligand binding in β-sandwiches.

## Materials and methods

Gene neighborhoods were determined using a custom script that uses completely sequenced genomes or whole genome shotgun sequences to derive a table of gene neighbors centered on a query gene. Then the BLASTCLUST program is used to cluster products in the neighborhood and establish conserved co-occurring genes. These conserved gene neighborhoods are then sorted as per a ranking scheme based on occurrence in at least one other phylogenetically distinct lineage ("phylum" in NCBI Taxonomy database), complete conservation in a particular lineage ("phylum") and physical closeness on the chromosome indicating sharing of regulatory -10 and -35 elements. Profile searches were conducted using the PSI-BLAST program [13] with a default profile inclusion expectation (E) value threshold of 0.01. Profile-profile comparisons were performed using the HHpred program [14]. HMM searches were conducted using the newly released HMMER3 program [15]. Multiple alignments were constructed using Kalign [16] followed by manual adjustments based on PSI-BLAST results. Protein secondary structure was predicted using a multiple alignment as the input for the JPRED program [17].

## Competing interests

The authors declare that they have no competing interests.

## Authors' contributions

LMI and LA were involved in the discovery process. RdeS and LA performed the analysis. RdeS and LA wrote the paper. The figures were prepared by RdeS and LMI. All authors have read and approved the final manuscript.

## Reviewers' comments

### Reviewer 1

M. Madan Babu, MRC Laboratory of Molecular Biology, University of Cambridge, UK.

In this manuscript, De Souza, Iyer and Aravind report the characterization of a family of proteins which are related to the *Anabena *sensory rhodopsin transducer. Through a rigorous computational analysis the authors first identified homologs of the ASRT gene in several bacteria that lack a sensory rhodopsin. Through a combination of genomic context and structural analysis, the authors report that this domain is likely to bind to small molecules, which are most likely to be sugars. In addition, the authors identify a subset of such proteins that are predicted to be secreted or membrane associated via lipid modification in several actinobacteria species, which also includes certain pathogens. Additional file 1 is comprehensive and provides a good substrate for experiments. In short, this is an excellent study and I would support publication of this work in Biology Direct upon minor revision:

1. It is currently unclear how the authors identified secreted proteins and the membrane associated proteins. Could the authors clarify this point, please? In addition, it would be good to see a comment on the functional significance of secreting sugar binding proteins rather than having them membrane associated.

### Authors' response

Secreted proteins where identified based on detection of an amino-terminal signal peptide by the program SignalP, followed by manual inspection. Transmembrane regions were detected using TMHMM. Except for the *Frankia sp*. homolog, which is fused to glycosyltransferase, all proteins with signal peptides (dyads and tetrads) did not have transmembrane regions and, therefore, are probably secreted. The functional significance of secreting sugar-binding proteins: these sugar binding domains in the extracellular proteins are likely to provide a means for associating with the sugar moieties in extracellular biopolymers such as peptidoglycans and other glycans present in surface capsules and gums.

### Reviewer 2

Mark A. Ragan, Institute for Molecular Bioscience, The University of Queensland

Brisbane, Australia.

I support the publication of your manuscript "The Anabaena sensory rhodopsin transducer defines a novel superfamily of prokaryotic small-molecule binding domains", by RF De Souza, LM Iyer and yourself, in Biology Direct as a Discovery Note.

## Supplementary Material

Additional file 1 **Supplementary material**. Material and methods and a complete list of conserved gene neighborhoods and comprehensive alignment of the: . Click here for file
