# Supplementary material for: The Anabaena sensory rhodopsin transducer defines a novel superfamily of prokaryotic small-molecule binding domains
Source: Biol Direct. 2009 Aug 14;4:25. doi: 10.1186/1745-6150-4-25 (PMC2739507; doi:10.1186/1745-6150-4-25)
Supplement: Additional file 1 — Supplementary material. Material and methods and a complete list of conserved gene neighborhoods and comprehensive alignment of the: . [file 1745-6150-4-25-S1.html]

SUPPLEMENTARY MATERIAL FOR: The Anabaena sensory rhodopsin transducer defines a novel superfamily of prokaryotic small-molecule binding domains 


  
**SUPPLEMENTARY MATERIAL   
 The Anabaena sensory rhodopsin transducer defines a novel superfamily of prokaryotic small-molecule binding domains**  
  
Robson F. de Souza,
Lakshminarayan M. Iyer,
and L. Aravind\*
  


\* *Address for correspondence: L. Aravind (aravind@mail.nih.gov)*  
  
 *National Center for Biotechnology Information, National Library of Medicine, National Institutes of Health, Bethesda, MD 20894, USA*  
  
  
  

|  |  |  |
| --- | --- | --- |
|  | ---     The Anabaena sensory rhodopsin transducer (ASRT) is a small protein that has been claimed to function as a signaling molecule downstream of the cyanobacterial sensory rhodopsin. However, orthologs of ASRT have been detected in several bacteria that lack rhodopsin, raising questions about the generality of this function. Using sequence profile searches we show that ASRT defines a novel superfamily of beta-sandwich fold domains. Through contextual inference based on domain architectures and predicted operons and structural analysis we present strong evidence that these domains bind small-molecules, most probably sugars. We propose that the intracellular versions like ASRT probably participate as sensors that regulate a diverse range of sugar metabolism operons or even the light sensory behavior in Anabaena by binding sugars or related metabolites. We also show that one of the extracellular versions define a predicted sugar-binding structure in a novel cell-surface lipoprotein found across actinobacteria, including several pathogens such as *Tropheryma*, *Actinomyces* and *Thermobifida*. The analysis of this superfamily also provides new data to investigate the evolution of carbohydrate binding modes in beta-sandwich domains with very different topologies.  ---  1. Materials and Methods - Comprehensive multiple alignment of the ASRAH domain - Domain architectures and gene neighborhoods of all ASRAH containing genes - List of species abbreviations      ---   **MATERIALS AND METHODS**  Profile-based searches were conducted using the PSI-BLAST and HMMER (Eddy, 1998) programs. PSI-BLAST (Altschul, et al., 1997) searches were performed against the nonredundant (NR) database of protein sequences and the non-redundant database of environmental sequences (National Center for Biotechnology Information [NCBI], NIH, Bethesda, MD, USA) with either a single sequence or an alignment used as query, with the default profile inclusion expectation (e) value set to a threshold of 0.01. Most searches were iterated until convergence. A statistical correction for compositional bias was used to reduce false positives (Schaffer, et al., 2001). In order to exhaustively recover all orthologs of the ASRAH domain, sequences recovered by each of these searches were further used as queries for PSI-BLAST searches and independent HMM-HMM profile alignment searches were also performed against a non-redundant version of the PDB database using HHpred (Sodding, 2005; Sooding et al. 2005). A single linkage clustering of the retrived proteins was then obtained using the BLASTCLUST program (ftp://ftp.ncbi.nih.gov/blast/documents/blastclust.html). A comprehensive multiple alignment was generated using the KALIGN program (Lassmann and Sonnhammer, 2005) and was further adjusted manually based on PSI-BLAST results, secondary structure predictions and multiple alignments of individual families. Protein secondary structure was predicted using the JPRED program (Cuff, et al., 1998) that uses information extracted from a PSSM, HMM, and the input seed alignment. The in-house TASS package (Anantharaman V, Balaji S, Aravind L; unpublished) was used for the automation of all large-scale sequence analysis procedures, including domain architectures and gene neighbourhood analysis. The pymol was used to prepare and analyze *Thermotoga*'s ASRAH homolog PDB structure. References   - Altschul, S.F., Madden, T.L., Schaffer, A.A., Zhang, J., Zhang, Z., Miller, W. and Lipman, D.J. (1997) Gapped BLAST and PSI-BLAST: a new generation of protein database search programs, Nucleic Acids Res, 25, 3389-3402. - Cuff, J.A., Clamp, M.E., Siddiqui, A.S., Finlay, M. and Barton, G.J. (1998) JPred: a consensus secondary structure prediction server, Bioinformatics, 14, 892-893. - Eddy, S.R. (1998) Profile hidden Markov models, Bioinformatics, 14, 755-763. - Finn, R.D., Mistry, J., Schuster-Bockler, B., Griffiths-Jones, S., Hollich, V., Lassmann, T., Moxon, S., Marshall, M., Khanna, A., Durbin, R., Eddy, S.R., Sonnhammer, E.L. and Bateman, A. (2006) Pfam: clans, web tools and services, Nucleic Acids Res, 34, D247-251. - Lassmann, T. and Sonnhammer, E.L. (2005) Kalign--an accurate and fast multiple sequence alignment algorithm, BMC Bioinformatics, 6, 298. - Letunic, I., Copley, R.R., Pils, B., Pinkert, S., Schultz, J. and Bork, P. (2006) SMART 5: domains in the context of genomes and networks, Nucleic Acids Res, 34, D257-260. - Schaffer, A.A., Aravind, L., Madden, T.L., Shavirin, S., Spouge, J.L., Wolf, Y.I., Koonin, E.V. and Altschul, S.F. (2001) Improving the accuracy of PSI-BLAST protein database searches with composition-based statistics and other refinements, Nucleic Acids Res, 29, 2994-3005. - S�ding J. (2005) Protein homology detection by HMM-HMM comparison.                 Bioinformatics 21, 951-960. doi:10.1093/bioinformatics/bti125.- S�ding J, Biegert A, and Lupas AN. (2005) The HHpred interactive server for protein homology detection and structure prediction.                   Nucleic Acids Research 33, W244--W248 (Web Server issue). doi:10.1093/nar/gki40.  --- |  |

```
2. Comprehensive multiple alignment of the ASRAH domain
Please click on the gene name to access the corresponding protein sequence.  

    


---


3. Domain architectures and gene neighborhoods of all ASRAH containing genes
```

You may click on the gene glyph to access the corresponding protein sequence.  
Genes without similarity to known domains or previously annotated genes are colored gray and have their names starting with an interrogation mark ('?')  
   


---

4.List of species abbreviations

|  |  |
| --- | --- |
| Aaro | *Aromatoleum aromaticum* |
| Aaur | *Arthrobacter aurescens* |
| Acel | *Acidothermus cellulolyticus* |
| Achl | *Arthrobacter chlorophenolicus* |
| Adeh | *Anaeromyxobacter dehalogenans* |
| Aodo | *Actinomyces odontolyticus* |
| Asp. | *Anaeromyxobacter sp.* |
| Asp. | *Arthrobacter sp.* |
| Asp. | *Azoarcus sp.* |
| Bado | *Bifidobacterium adolescentis* |
| Bang | *Bifidobacterium angulatum* |
| Bani | *Bifidobacterium animalis* |
| Bbif | *Bifidobacterium bifidum* |
| Bbre | *Bifidobacterium breve* |
| Bcat | *Bifidobacterium catenulatum* |
| Bden | *Bifidobacterium dentium* |
| Bgal | *Bifidobacterium gallicum* |
| Blin | *Brevibacterium linens* |
| Bpse | *Bifidobacterium pseudocatenulatum* |
| Cagg | *Chloroflexus aggregans* |
| Cmic | *Clavibacter michiganensis* |
| Csac | *Caldicellulosiruptor saccharolyticus* |
| Dthe | *Dictyoglomus thermophilum* |
| Dtur | *Dictyoglomus turgidum* |
| Faln | *Frankia alni* |
| Fsp. | *Frankia sp.* |
| Gsp. | *Geobacillus sp.* |
| Haur | *Herpetosiphon aurantiacus* |
| Jsp. | *Janibacter sp.* |
| Mact | *marine actinobacterium* |
| Nham | *Nitrobacter hamburgensis* |
| Nmag | *Natrialba magadii* |
| Noce | *Nitrosococcus oceani* |
| Nsp. | *Nocardioides sp.* |
| Rcas | *Roseiflexus castenholzii* |
| Rgna | *Ruminococcus gnavus* |
| Rsal | *Renibacterium salmoninarum* |
| Rsp. | *Roseiflexus sp.* |
| Scel | *Sorangium cellulosum* |
| Scla | *Streptomyces clavuligerus* |
| Sgri | *Streptomyces griseus* |
| Smal | *Stenotrophomonas maltophilia* |
| Smed | *Sinorhizobium medicae* |
| Snod | *Streptomyces nodosus* |
| Ssp. | *Streptomyces sp.* |
| Ssvi | *Streptomyces sviceus* |
| Tnea | *Thermotoga neapolitana* |
| Tpet | *Thermotoga petrophila* |
| Twhi | *Tropheryma whipplei* |

---
